# Fmoc-PEG Coated Single-Wall Carbon Nanotube Carriers by Non-covalent Functionalization: An Experimental and Molecular Dynamics Study

**DOI:** 10.3389/fbioe.2021.648366

**Published:** 2021-05-14

**Authors:** Yesim Yeniyurt, Sila Kilic, Ö. Zeynep Güner-Yılmaz, Serdar Bozoglu, Mehdi Meran, Elif Baysak, Ozge Kurkcuoglu, Gurkan Hizal, Nilgun Karatepe, Saime Batirel, F. Seniha Güner

**Affiliations:** ^1^Department of Chemical Engineering, Istanbul Technical University, Istanbul, Turkey; ^2^Energy Institute, Renewable Energy Division, Istanbul Technical University, Istanbul, Turkey; ^3^Department of Bioengineering, Faculty of Engineering and Natural Sciences, Üsküdar University, Istanbul, Turkey; ^4^Department of Chemistry, Istanbul Technical University, Istanbul, Turkey; ^5^Department of Medical Biochemistry, Faculty of Medicine, Marmara University, Istanbul, Turkey; ^6^Sabancı University Nanotechnology Research and Application Center (SUNUM), Sabancı University, Istanbul, Turkey

**Keywords:** carbon nanotubes, molecular dynamic simulation, Fmoc-protected amino acids, PEG, non-covalent modification, nanocarrier (nanoparticle)

## Abstract

Due to their structural characteristics at the nanoscale level, single-walled carbon nanotubes (SWNTs), hold great promise for applications in biomedicine such as drug delivery systems. Herein, a novel single-walled carbon nanotube (SWNT)-based drug delivery system was developed by conjugation of various Fmoc-amino acid bearing polyethylene glycol (PEG) chains (Mw = 2,000, 5,000, and 12,000). In the first step, full-atom molecular dynamics simulations (MD) were performed to identify the most suitable Fmoc-amino acid for an effective surface coating of SWNT. Fmoc-glycine, Fmoc-tryptophan, and Fmoc-cysteine were selected to attach to the PEG polymer. Here, Fmoc-cysteine and -tryptophan had better average interaction energies with SWNT with a high number of aromatic groups, while Fmoc-glycine provided a non-aromatic control. In the experimental studies, non-covalent modification of SWNTs was achieved by Fmoc-amino acid-bearing PEG chains. The remarkably high amount of Fmoc-glycine-PEG, Fmoc-tryptophan-PEG, and Fmoc-cysteine-PEG complexes adsorbed onto the SWNT surface, as was assessed via thermogravimetric and UV-vis spectroscopy analyses. Furthermore, Fmoc-cysteine-PEG_5000_ and Fmoc-cysteine-PEG_12000_ complexes displayed longer suspension time in deionized water, up to 1 and 5 week, respectively, underlying the ability of these surfactants to effectively disperse SWNTs in an aqueous environment. *In vitro* cell viability assays on human dermal fibroblast cells also showed the low cytotoxicity of these two samples, even at high concentrations. In conclusion, synthesized nanocarriers have a great potential for drug delivery systems, with high loading capacity, and excellent complex stability in water critical for biocompatibility.

## Introduction

Among nanocarriers, single-walled carbon nanotubes (SWNTs) are currently one of the most investigated materials, and a great deal of effort is devoted to expanding their biomedical applications, particularly for treatment (Mehdipoor et al., 2011; Sheikhpour et al., 2017) and imaging (Yudasaka et al., 2017) purposes. Despite the appreciable progress, the toxicity of pristine SWNTs poses a great obstacle for their usage in biomedical applications. To overcome this, SWNTs need to be functionalized with biocompatible agents. Functionalization of SWNTs with biopolymers (Fakurazi et al., 2018), suitable ligands (Sadjadi et al., 2018), or functional groups (Mohamed et al., 2020) assists in site-specific administration of a drug and site-specific recognition of the nanocarrier (Adeli et al., 2013). Non-covalent functionalization possesses advantages over the other functionalization methods in terms of structural and functional properties of both nanotubes and biomolecules, exploiting mostly π-π interactions (Chen et al., 2001; Zorbas et al., 2005; Pender et al., 2006), as well as hydrophobic interactions, and electrostatic adsorption (Bilalis et al., 2014). Such interactions between carbon nanotubes (CNTs) and polymers eventually lead to the separation of individual CNTs and thus better dispersion, since they are strong enough to obviate the intertubular van der Waals interaction forces (Basheer et al., 2020). Intermolecular overlap of p-orbitals in conjugated systems leading to strong π-π interactions increases the dispersion of SWNTs in an aqueous medium as reported by numerous studies (Nepal et al., 2005; Ortiz-Acevedo et al., 2005; Geckeler, 2007; Kim et al., 2007; Liang et al., 2017). Molecules such as pyrene and its derivatives have been reported to non-covalently bind to the surfaces of SWNTs by establishing π-π stacking interaction due to their phenyl rings (Nakashima et al., 2002; Petrov et al., 2003; Fifield et al., 2004; Nakashima, 2006; Feazell et al., 2007). Similarly, coating the surface of CNTs using biomolecules such as peptides, proteins, and DNA through π-π interactions has been discussed in detail in the literature (Sun, 2004; Antonucci et al., 2017; Lu et al., 2018; Demirer et al., 2019).

Polyethylene glycol (PEG) is a non-immunogenic, non-antigenic, and FDA-approved polymer, which has been widely employed as a coating to improve the biocompatibility of various molecules (Verhoef and Anchordoquy, 2013), including SWNTs (Meran et al., 2018). It preserves the graphitic surface and intrinsic nature of SWNT (Wulan and Salsabila, 2020), as well as reduces the formation of agglomeration of nanotubes by providing them dispersibility and stability in physiological conditions (Premkumar et al., 2012; Suk et al., 2016). Furthermore, PEG may prolong the circulation time of a protein or nucleic acids, enhance the aqueous solubility of drugs, protect the cargo against *in vivo* biological inactivation by proteolysis, and reduce the immunogenicity of biopharmaceuticals (Verhoef and Anchordoquy, 2013; Lee, 2015). Aromatic molecules such as pyrene, porphyrins, and their derivatives are operated to increase the binding efficiency of PEGs as they can interact with the sidewalls of SWNTs through aromatic interactions (Cousins et al., 2009; Backes and Hirsch, 2010; Zhang et al., 2013; Naqvi et al., 2020). Interaction between aromatic groups involves aromatic donors and acceptors, where off-center parallel stacking, edge-to-face stacking, and face-centered stacking can be favored according to their aromatic quadrupole moments (Martinez and Iverson, 2012). Similarly, the intrinsic hydrophobicity and aromatic nature of the Fmoc unit reinforce the hydrophobic and aromatic interactions of the fluorenyl rings (Fleming and Ulijn, 2014; Tao et al., 2016). Amino acids and peptides modified with Fmoc display self-assembly properties, and are used in drug delivery systems for antibiotics and therapeutic agents (Wang and Chau, 2011; Guilbaud-Chéreau et al., 2019; Martin and Thordarson, 2020). Fmoc modified amino acids and peptides ensure enhanced hydrophobicity, thus stabilize the overall conformation or allow contrast agents that can be employed for magnetic resonance imaging (Zhang et al., 2019). In a study investigating an alternative treatment for breast cancer, the protein annexin V was operated as a targeting component. A recent approach was employed to ensure strong bonding of the amino groups of annexin V to the SWNT surface, and Fmoc-amine-PEG-succinimidyl carboxymethyl ester was used as the anchor molecule (Neves, 2012). Wang and Chau (2011) reported self-assembling Fmoc-tris (2-aminoethyl) amine (TAEA) and Fmoc-phenylalanine-tyrosine nano-carriers, which are well-tolerated by cells. In another study, multiwall CNT/Fmoc–Tyr–amine nanoparticles displayed reduced protein-binding properties, low cytotoxicity, and immune response (Zhang et al., 2010). Similarly, Li et al. (2009) examined the interaction of graphene and CNT surfaces with Fmoc modified using tryptophan, glycine, tyrosine, phenylalanine, and histidine. The adsorption experiments and kinetic calculations highlighted Fmoc-tryptophan with its highest affinity to the aromatic carbon surfaces among others (Li et al., 2009). Backes and Hirsch reported that the dispersibility of MWNTs in water was significantly improved with the covalently attached cysteine molecules when compared to pristine MWNTs (Backes and Hirsch, 2010).

Here, we investigated the non-covalent modification of SWNTs with PEG chains bearing Fmoc-amino acids. To determine suitable Fmoc-amino acids with the required capability to adsorb on the nanotube surface, full-atom molecular dynamics simulations in explicit water were conducted. Six different Fmoc-amino acid-coated SWNT complexes were constructed, in which five aromatic amino acids, tryptophan, tyrosine, histidine, phenylalanine, and S-trityl-L-cysteine, and non-aromatic glycine were investigated. Based on their extent of interactions with SWNT, three Fmoc-amino acids were chosen to experimentally explore the non-covalent functionalization of SWNTs: Fmoc-tryptophan, Fmoc-cysteine, and Fmoc-glycine as non-aromatic control.

The SWNT length and the molecular weight of the PEG chain are other critical parameters for the SWNT coating. In our previous study, we demonstrated that short SWNTs can be more effectively coated with the PEG chains when compared to long SWNTs. Indeed, in line with the *in vitro* studies, short SWNTs wrapped with long PEG chains showed low cytotoxicity (Meran et al., 2018). Therefore, in this study, we used short SWNTs synthesized in our previous work. We also demonstrated that pyrene attached long PEG chains with a molecular weight of 12,000 (PEG_12000_) provide more effective SWNT coating to achieve *in vitro* biocompatibility when compared to shorter chains (PEG_2000_ and PEG_5000_) (Zhang et al., 2010). While the anchor molecule changes, the interaction behavior of different molecular weighted PEG chains may be strikingly different. For this reason, three different molecular weights of PEG (Mw = 2,000, 5,000, and 12,000) were employed to understand the effect of PEG chain length on SWNT coating efficiency. This study explains how to functionalize SWNTs with Fmoc-amino acid bearing PEGs. It also brings a new perspective to the non-covalent functionalization of SWNTs, using a highly effective biocompatible coating that significantly improves the water suspension time and achieves low cytotoxicity on human fibroblast cells. Synthesized SWNT-based nanoparticles are highly promising for long blood circulation time in *in vivo* applications.

## Computational and Experimental Methods

### Molecular Dynamics Simulations

We investigated six N-(fluorenyl-9-methoxycarbonyl) (Fmoc) attached amino acids, which are glycine (G), phenylalanine (F), histidine (H), tryptophan (W), tyrosine (Y), and S-trityl-L-cysteine (C), to understand their extent of interactions with the SWNT surface. Fmoc-X-OH structures were downloaded from the PubChem database. Before the simulations, they were energetically minimized using the smart algorithm (a combination of steepest descent, ABNR, and quasi Newton-Raphson methods) of Materials Studio with an energy tolerance of 0.001 kcal/mol. In all calculations, the COMPASS force field was used (Sun, 1998). A single-walled carbon nanotube (SWNT) with (6,6) chirality is generated with the *Build* module of Materials Studio. The SWNT had a diameter of 8.14 Å and a length of 24.6 Å, which corresponded to the SWNTs used in the experiments.

Three different computational approaches were consecutively implemented to determine the most suitable Fmoc-amino acid to coat the SWNTs; (1) Simulated annealing simulations to obtain the most energetically favorable configurations of Fmoc-amino acids on the SWNT surface, (2) full-atom molecular dynamics (MD) simulations in explicit water coupled with energy minimization to sample around the energetically most favorable configurations obtained from the first step, and (3) 10 ns long full-atom MD simulations of promising Fmoc-amino acids adsorbed on the SWNT determined from the second step.

Simulated annealing simulations using the Monte Carlo technique were performed with the *Adsorption Locator* module. The temperature was gradually decreased from 10^5^ K to 100 K so that adsorbates can effectively scan various configurations on the SWNT at high temperature and were directed toward the low energy pits of the energy surface with decreasing temperature. Simulations were carried with 5 cycles, where 10^5^ steps/cycle of Monte Carlo were used to adsorb the molecules on the SWNT surface. For electrostatic and van der Waals interactions, a cut-off distance of 15.5 Å was taken. Here, multiple Fmoc-amino acid molecules were adsorbed on the SWNT surface; seven adsorbates were determined to sufficiently cover the SWNT surface for all cases.

Then, seven Fmoc-amino acids adsorbed on the SWNT were solvated with explicit water fixing the density at 1.2 g.cm^–3^ matching the experimental conditions. The system was energetically minimized as described above and then equilibrated using a 20 ps long MD simulation with NVT ensemble at 298 K while fixing the SWNT. Berendsen thermostat with a decay constant of 0.1 ps was employed to keep the temperature constant. Electrostatic interactions were computed using the Ewald summation technique. Following the equilibration, the aqueous system was re-conditioned with Quench MD simulations of Materials Studio. Quench MD simulation is a classical MD simulation run between geometry optimizations, together called quench, applied with the desired frequency, which was set as 100 ps. Thus, an effective configurational sampling of Fmoc-amino acid – SWNT complexes in water was achieved. MD simulations were carried for 1 ns, which was found adequate for the Fmoc-amino acid derivatives on SWNT. Based on the energetically minimized complexes, interaction energies E_*int*_ (kcal.mol^–1^) between the Fmoc-amino acid molecules and SWNTs were calculated by,

(1) $$E_{i\,n\,t}=E_{c\,o\,m\,p\,l\,e\,x}-\left(E_{S\,W\,N\,T}+E_{F\,m\,o\,c-a\,m\,i\,n\,o\,a\,c\,i\,d}\right)$$

Here, *E*_*complex*_ is the total energy of seven Fmoc-amino acid molecules adsorbed on SWNT, *E*_*SWNT*_ and *E*_*Fmoc–amino acid*_ are the total energies of the SWNT and the adsorbates, respectively.

Finally, 10 ns long full-atom classical MD simulations of three Fmoc derivatives, namely -G, -W, and -C adsorbed on SWNTs were further investigated in the explicit solvent under the same conditions of the quench MD simulations.

### Experimental Studies

#### Materials

Polyethylene glycol monomethyl ether with 2,000, 5,000, and 12,000 g.mol^–1^ molecular weight (PEG_2000_, PEG_5000,_ and PEG_12000_, respectively) were purchased from Sigma-Aldrich. Fmoc-glycine-OH, Fmoc-tryptophan-OH, and Fmoc-cysteine (Trt)-OH were also obtained from Sigma-Aldrich. Dichloromethane (DCM) was purchased from Sigma-Aldrich and distilled over P_2_O_5_ before usage. Tetrahydrofuran (THF; 99.8%, J.T. Baker) was dried and distilled over benzophenone–Na. Diethyl ether, dimethylaminopyridine (DMAP), and *N, N’*-dicyclohexylcarbodiimide (DCC) were all purchased from Sigma-Aldrich and used as received without further purification.

#### SWNTs Synthesis

Single-walled carbon nanotubes were synthesized using the fluidized-bed chemical vapor deposition (CVD) (Kim et al., 2007) of acetylene (C_2_H_2_) on magnesium oxide (MgO) powder impregnated with an iron nitrate [Fe(NO_3_)_3_⋅9H_2_O] solution. The CVD apparatus consisted of a vertical furnace and a quartz glass tube with a diameter of 3 cm in the middle of a quartz filter. A magnesium oxide (100 m^2^⋅g^–1^) supported iron oxide powder produced by impregnation in an iron nitrate ethanol solution was used as a precursor powder. To obtain a precursor with a MgO-to-Fe weight ratio of 5%, MgO suspended in ethanol and iron nitrate (dissolved in 100 mL of ethanol) was stirred together and sonicated for 20 min to homogenize the mixture. Afterward, the precursor was dried and ground into a fine powder. For one deposition, typically 0.6 g of precursor powder was filled in the quartz tube, and the atmosphere was purged with argon for 5 min. Then, the furnace was heated to the synthesis temperature (800°C). By heating the precursor powder, iron oxide clusters were formed because of the thermal decomposition of the iron nitrate at 125°C. The synthesis was started with the introduction of acetylene mixed with argon for 30 min. After synthesis, SWNTs were purified with 6M HNO_3_ for 3 h.

#### Preparation of Fmoc-PEGs

PEG_2000_ (3.0 g, 1.5 mmol) was dissolved in CH_2_Cl_2_ (30 ml). Then, Fmoc-glycine-OH (0.67 g, 2.25 mmol), Fmoc-tryptophan-OH (0.96 g, 2.25 mmol), or Fmoc-cysteine (Trt)-OH (1.32 g, 2.25 mmol) dissolved in 10 ml of THF and DMAP (0.122 g, 1.0 mmol) were added into the mixture. DCC (0.56 g, 2.7 mmol) dissolved in 10 mL of CH_2_Cl_2_ was added to the mixture. The reaction mixture was stirred at room temperature for 48 h, then concentrated and precipitated in diethyl ether. The polymer was isolated by filtration and dried under the vacuum for 24 h. The same procedure was followed for PEG_5000_ and PEG_12000_ to obtain Fmoc-PEGs. For all samples, calculations were done for 3 grams of PEG and their molar equivalents.

#### Functionalization of SWNTs With Fmoc-PEGs

Single-walled carbon nanotubes (50 mg) and Fmoc-PEG_2000_ (0.25 g) were added to a flask containing 50 mL of dry THF. The mixture was sonicated in an ultrasonic bath for 30 min and then stirred at room temperature for 48 h. The final mixture was filtered (Sartorius, PTFE; pore size, 0.2 μm) to remove non-attached Fmoc-PEGs. The remaining solid on the filter was further washed using fresh THF. The obtained black powder was dried under the vacuum for 24 h. The same procedure was employed to obtain the other nanocarriers.

In addition, we repeated the above procedure with SWNTs (50 mg) and Fmoc-amino acid (0.32 g) to obtain SWNT/Fmoc-amino acid complexes to assess the MD simulations. To determine the Fmoc adsorption capacity of SWNTs and reveal the correlation between adsorption amount of functional monomer and dispersion behavior for coated SWNTs, the dynamic behavior of Fmoc-amino acid loading was investigated using a UV-vis spectrophotometer. First, Fmoc-glycine-OH, Fmoc-tryptophan-OH, or Fmoc-cysteine (Trt)-OH (0.16 g) was dissolved in THF (25 mL). SWNTs (0.025 g) were dispersed in this solution and incubated with an orbital shaker at 28°C. Then, the solution was filtered through a Fluoropore Membrane Filter with 0.45 μm pore size, and the absorbance of the collected solution was determined using a calibration curve.

#### Characterization

^1^H NMR (500 MHz) spectra were recorded in CDCl_3_, using Agilent Technologies Cary 630 FT-IR instrument over the range 4,000–650 cm^–1^. UV–vis spectra were recorded on an Agilent Cary 100 spectrophotometer in CH_2_Cl_2_. Fluorescence spectra were obtained with an F-4500 spectrophotometer. Thermal gravimetric analyses (TGA) were performed on Perkin–Elmer Diamond TA/TGA in the temperature range of 30–800°C, with a heating rate of 10°C.min^–1^ under nitrogen. TEM images of SWNTs were obtained with a FEI-Tecnai-F20 transmission electron microscope. Raman spectra were recorded using Horiba Jobin-YVON HR 800 UV instrument.

Additionally, the dispersion of SWNTs and SWNT/Fmoc-amino acid complexes in water was monitored. For this purpose, pristine SWNTs and functionalized SWNTs (5 mg) were put in flasks, sonicated in an ultrasonic bath in 25 mL water. Then, the dispersion behavior was observed under the force of gravity, and flasks were photographed at specified times. The dispersion behavior was also monitored for all Fmoc-PEG functionalized SWNTs.

#### *In-vitro* Cell Viability Assay

Cytotoxicity of the samples was evaluated with MTT-assay. PCS-201-012 human dermal fibroblast cells (American Tissue Culture Collection, VA, United States) were cultured in RPMI 1640 supplemented with 10% Fetal Bovine Serum (FBS), 100 IU.mL^−1^ penicillin, and 100 μg.mL^−1^ streptomycin in a humidified 5% CO_2_ incubator at 37°C. The cells were seeded in 96-well plates. After 24 h of incubation with the samples, the medium from the wells was removed. 5 mg/ml MTT was added to each well. The plates were incubated for 3 h in 5% CO_2_ incubator. During incubation, living cells metabolized the MTT and blue formazan crystals were formed. The crystals were dissolved in DMSO. The absorbance of the suspensions was read at 590 nm in a microplate reader. The viabilities of the cells were presented as a percentage of the control values.

All experimental studies were performed at least three times and each concentration was done in triplicates to achieve independent experiments for the cell viability. Results are given as mean ± standard deviation (SD). In the evaluation of data, statistical analysis was done by two-way analysis of variance (ANOVA). Differences were considered significant if *p* ≤ 0.05.

## Results and Discussion

Before SWNT coating with Fmoc–amino acid–PEG chains, molecular interactions between SWNT and different Fmoc–amino acids were investigated using computational techniques to determine the most suitable anchor(s) to attach the hydrophilic chain ends. Accordingly, we investigated Fmoc-S-trityl-L-cysteine (C), Fmoc-phenylalanine (F), Fmoc-histidine (H), Fmoc-tryptophan (W), Fmoc-tyrosine (Y), and Fmoc-glycine (G), where the latter has hydrogen as a side chain. We first discuss the results obtained from the MD simulations, then concentrate on the experimental findings to show how an effective non-covalent coating of SWNTs was achieved.

### Selecting Fmoc-Amino Acids for SWNT Coating Using MD Simulations

Molecular interactions between the Fmoc-amino acids and SWNT are critical for an effective coating of the nanotube and preserving its biocompatibility during the application. For this reason, we focused not only on the interactions between Fmoc-amino acids and SWNTs but also on the interactions between multiple Fmoc-amino acids adsorbed on the nanotube surface.

**FIGURE 1:**
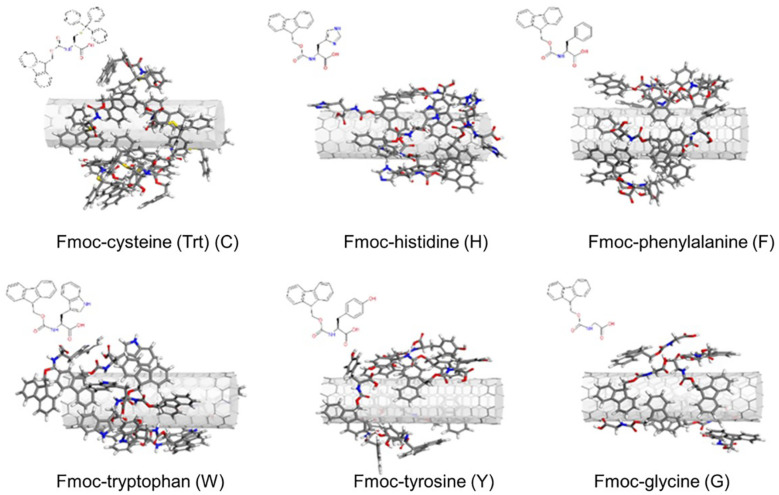
Configurations of the Fmoc – amino acids on the SWNT surface obtained with simulated annealing simulations.

Simulated annealing simulations enabled obtaining the lowest energy configurations of Fmoc – amino acids adsorbed on SWNT. Since a good surface coverage of the nanotube was aimed, we were able to adsorb seven Fmoc-amino acids on the SWNT surface, achieving a rather homogeneous distribution over the nanotube surface (Figure 1). As the number of adsorbates was increased, a second layer of Fmoc – amino acids was obtained on the nanotube especially for C. Therefore, the number of adsorbates was held to be seven for all systems to compare interaction energies of Fmoc derivatives with SWNT (6,6).

As expected, aromatic groups of Fmoc – amino acids established π – π interactions with the SWNT surface. Surface coverage by G molecules was poorer when compared to other derivatives since G lacks a bulky aromatic side chain. Nonetheless, all complexes offered plausible starting configurations for the MD simulations.

**FIGURE 2:**
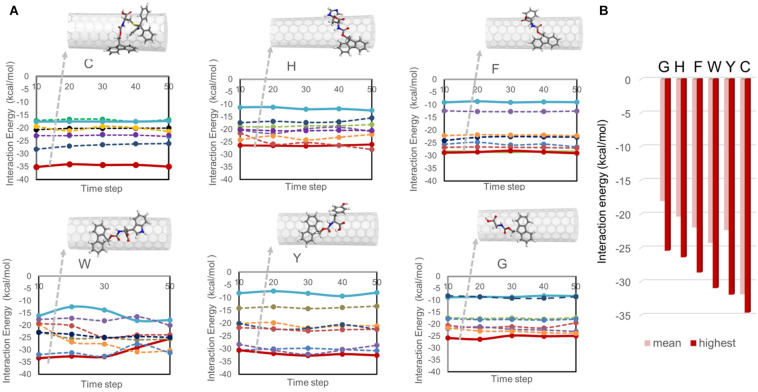
Interaction energies between Fmoc – amino acids and SWNT calculated from quench MD simulations. In **(A)**, the interaction energies are plotted with respect to the simulation trajectory. In **(B)**, the mean and highest interaction energies for seven Fmoc derivatives per system are shown.

The configurations obtained from simulated annealing simulations were then investigated using full-atom MD simulations coupled with energy minimization, referred to as quench MD. Seven Fmoc derivatives were allowed to sample the nanotube surface, and their interaction energies with the SWNT were calculated for the energetically minimized configurations. Figure 2A displays the interaction energy profiles for all cases. Each Fmoc – amino acid had a different amount of interaction with the SWNT, which changed during the sampling due to the dynamic nature of the system. Some Fmoc derivatives interacted with the SWNT surface both using Fmoc groups and amino acid side chains, and thus maximized the number of π-π interactions resulting in stronger interactions with the SWNT (red lines in Figure 2A). The snapshots for the configurations with the strongest interactions are shown on top of the plots. On the other hand, some Fmoc derivatives also established π-π interactions with each other and made stackings, which led to edge-to-face orientations of the aromatic groups over the nanotube. This was reflected as poor interaction energies (blue lines in Figure 2A). Here, W and C molecules had an interaction energy range of around −15 and −35 kcal.mol^–1^, distinguishing them from the others having better interaction energies overall.

Figure 2B displays the interaction energy values of the best poses of the Fmoc derivatives shown in Figure 2A and their mean values. In descending order, the highest interaction energies are −34.58 kcal.mol^–1^ for C, –31.92 kcal.mol^–1^ for Y, −30.95 kcal.mol^–1^ for W, −28.63 kcal.mol^–1^ for F, −26.38 kcal.mol^–1^ for H, and −25.41 kcal.mol^–1^ for G. On the other hand, there were seven copies of the Fmoc derivatives on the nanotube, which had different amounts of interactions based on their positions on the SWNT (Figure 2A). The average interaction energies are given in Figure 2B. Accordingly, C (−31.85 kcal.mol^–1^) and G (−18.03 kcal.mol^–1^) molecules set the upper and lower limits. Fmoc derivatives can be arranged according to their average interaction strengths with SWNT as C > W > Y > F > H > G. This trend is in line with a previous study investigating only one Fmoc – amino acid derivative adsorbed on (6,6) SWNT (Cousins et al., 2009).

**FIGURE 3:**
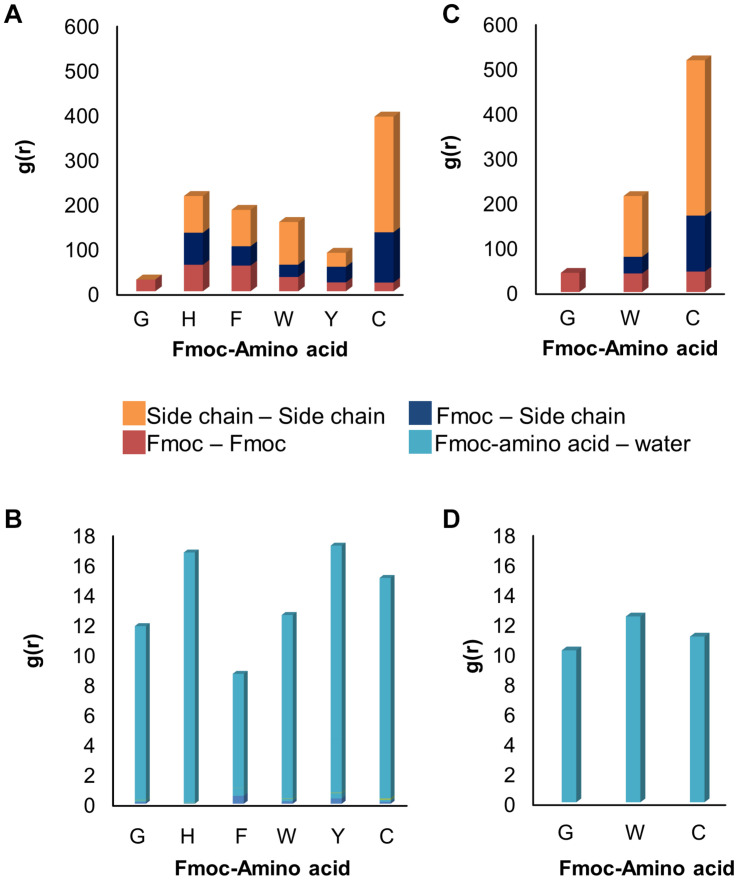
RDF results showing the extent of **(A)** π−π and **(B)** hydrogen bond interactions in 1 ns long quench MD simulations, and **(C)** π−π and **(D)** hydrogen bond interactions in 10 ns long classical MD simulations.

In addition, Fmoc – amino acids formed π-π interactions among themselves while maintaining their interactions with the SWNT surface. Figure 3A shows the extent of π-π interactions between different aromatic groups of Fmoc derivatives during the quench MD simulations sampling the most favorable energy configurations. Here, the interactions were quantified for Fmoc-Fmoc pairs, the Fmoc-aromatic side chain of the amino acid pairs, and aromatic side-chain – aromatic side-chain pairs. There were seven adsorbates in each system, where we analyzed the RDF plot for each interacting group of atoms. In Figure 3A, the magnitudes of the g(r) peaks are given for the π-π interactions. To attain a general picture of the system, magnitudes of g(r) peaks are displayed as a cumulative sum over seven adsorbates. Accordingly, C molecules made the highest number of interactions among themselves due to four aromatic groups on their side chains. These derivatives were able to make these interactions still attached to the SWNT surface, either directly or by stacking. To illustrate this, Supplementary Figures 1, 2 show the distance evolution between the nanotube surface and aromatic groups Fmoc and side chains, respectively. Both Fmoc and side chains of C molecules had the highest distance from the SWNT surface implying stacking of aromatic groups on the nanotube surface. Nonetheless, as C molecules are adsorbed on the SWNT surface, they do not desorb during the simulation. The latter observation was also made for the other molecules. The extent of π-π interactions between Fmoc derivatives is in decreasing order as – C, – H, – F, –W, – Y, and – G. G had a rather limited interaction with the other adsorbates on the SWNT due to its single aromatic group, Fmoc (Figure 3A).

The dispersion stability of SWNTs in water also depends on the extent of hydrogen bond interactions between Fmoc – amino acids and water molecules. Figure 3B displays the RDF results that summarize the strength of g(r) peaks at ∼2.0 Å indicating hydrogen bonds between water and amine or carboxylic groups. The strengths of g(r) peaks changed according to the type of amino acids. Fmoc – F molecules had the least amount of hydrogen bond interactions with water implying a less stable complex in the aqueous environment. Fmoc – H, – Y, and – C had more stable interactions with water suggesting a good dispersion of the SWNT complexes in water, with the condition that aromatic groups maintain their π-π interactions with the nanotube.

Based on these results, three Fmoc derivatives with the lowest (G) and highest interaction energies (W, C), were further investigated using classical MD simulations. During the simulations, adsorbed Fmoc derivatives did not desorb from the SWNT surface (Supplementary Figure 3), although they interacted with each other (Figure 3B). With the increasing number of aromatic groups in their side chains, Fmoc derivatives had higher amounts of π-π interactions with the other adsorbates. Both quench and classical MD simulations pointed to the same findings; the most effective SWNT coating was expected for C and W while the poor coating was anticipated for G. Additionally, all surfactants had similar extents of hydrogen bond interactions with water (Figure 3D). Results suggested that Fmoc-C would lead to better nanotube stability in the water when compared to W and G since it seemed to better adsorb on SWNT (Figure 3C).

### Synthesis of SWNTs

TEM, being the highest resolution visualization technique, is typically capable of imaging CNT samples up to atomic resolution. TEM images of the synthesized sample are shown in Supplementary Figure 4A. It was evident that the structures synthesized by the chemical vapor deposition method were CNTs. The dark regions of the figure was plausibly due to the impurities existing within the synthesized material. TEM images of the samples indicated that SWNTs were synthesized at 800°C.

Raman spectroscopy is a powerful technique for the characterization of the structure of CNTs. Supplementary Figure 4B shows Raman spectra for synthesized carbon deposit excited by 633 nm laser. Two different spectra of CNTs were observed at the G band (around 1,570 cm^–1^) and D band (around 1,300 cm^–1^). Here, the intensity of the G band is higher than D band. The intensity ratio of D and G band (ID/IG) expresses the quality of CNTs. A higher ratio refers to a higher amorphous carbon content and defect formation. The intensity ratio of G and D bands of the synthesized CNT sample was found as 0.69, which indicated that CNTs had a low amorphous carbon content and defects. The spectrum in the RBM band observed for the sample, is a characteristic of SWNT and showed that the nanotube diameter is below 2 nm. Radial breathing mode (RBM) band can be used to calculate the mean diameter of SWNTs by,

(2) $$\omega \,\left(c\,m^{-1}\right)=A/d\,\left(n\,m\right)+B\,\left(c\,m^{-1}\right)$$

where; ω is RBM frequency (245.6 cm^–1^), *A* and *B* are constants (*A* = 223 cm^–1^/nm, *B* = 10 cm^–1^) and *d* is the diameter of SWNT. The calculated mean diameter of the synthesized SWNT sample was found as 0.94 nm.

### Coating SWNTs With Fmoc-Amino Acids

**FIGURE 4:**
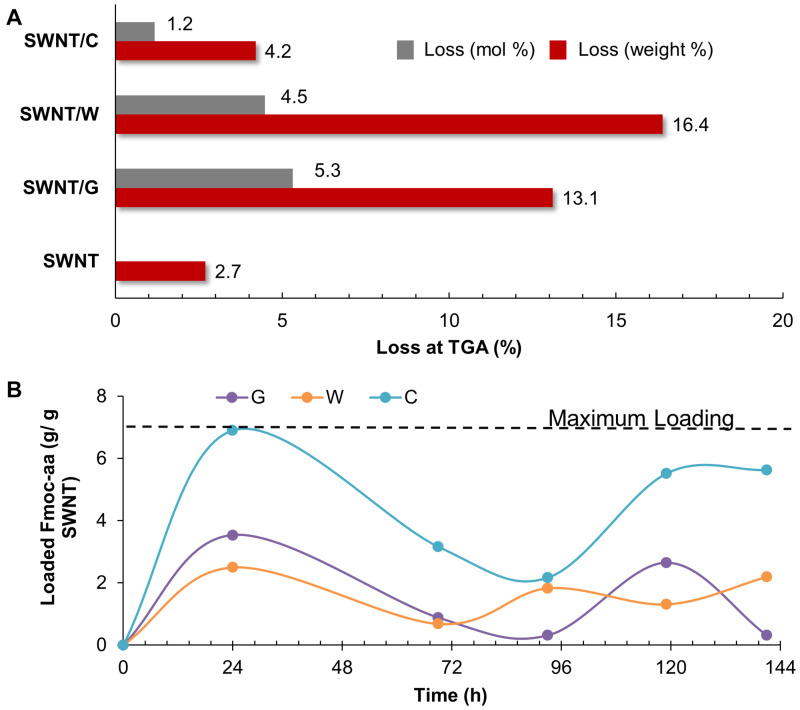
**(A)** Thermogravimetric results, and **(B)** adsorption profiles of SWNTs coated with Fmoc-cysteine (C), Fmoc-tryptophan (W), and Fmoc-glycine (G),

Before incorporating PEG chains into the nanoparticles, C-, W-, and G-functionalized SWNTs were investigated via TGA (Supplementary Figure 5). The weight loss at 800 °C gave the weight percentage of the organic matter, Fmoc-amino acids in our case, as explained in literature (Petrov et al., 2003; Bilalis et al., 2014). Figure 4A depicts the mole and weight loss percentages for the functionalized SWNTs calculated from TGA curves. The weight loss of around 2.7% for the synthesized SWNTs was possibly due to the desorption of moisture and/or the decomposition of functional groups (carboxyl etc.) formed on the surface of SWNTs during nitric acid treatment. To compare the coating amounts, SWNT weight loss (2.7%) was reduced from the weight loss of Fmoc coated SWNT samples. The highest amount of coating was achieved with W while C had minimum weight losses. To compare experimental findings with the computational results, the losses in moles of Fmoc derivatives were calculated as percentages, and all discussion will be thus made over this calculation. TGA analysis indicated that the number of G molecules coating the SWNT was slightly higher when compared to the others with extra aromatic groups on their side chains. C, with the highest amount of aromatic groups, interestingly seemed to adsorb on SWNT less. There may be two major explanations for this intriguing observation. First, with the increasing number of aromatic groups in their side chains, in the order of G < W < C, Fmoc derivatives had a higher amount of π-π interactions including stackings with the other adsorbates as suggested by the MD simulations (Figures 3A,B). This in turn can promote the release of C and W molecules that tether the SWNT surface by stacking interactions, during the washing process. On the other hand, Figure 4A shows that the attachment of Fmoc-amino acids to the SWNT surface is inversely proportional to their molecular weights or their excluded volumes. MD simulation snapshots in Figure 2A display that the excluded volume of G is lower than those of C and W, leading to more adsorbed G molecules on the SWNT surface. We then calculated the maximum theoretical amount of Fmoc-amino acids forming a single layer coating on SWNT using simulated annealing simulations with the Monte Carlo method. The SWNT (6,6) accommodated 7 C, 8 W, or 9 G molecules at maximum, while it could not encapsulate Fmoc derivatives. These amounts corresponded to 1.41 g C/g SWNT, 1.17 g W/g SWNT, and 0.92 g G/g SWNT.

We also determined the adsorption profiles of Fmoc molecules and the adsorption capacity of SWNTs using loading experiments. For this purpose, G, W, or C was dissolved in THF to prepare a stock solution for each Fmoc-amino acid. Then, a set of Fmoc-amino acid solution was prepared from the stock solution in various concentrations. The maximum wavelength was determined with a typical wavelength scan for each solution between 290 and 300 nm, and the data obtained was checked with those reported in the literature (Oliverio et al., 2014; Tao et al., 2015; Eissler et al., 2017; Das Mahapatra et al., 2019; Fidecka et al., 2020; Szczepa et al., 2020). We used the peak at 300 nm for the calculation of the loading of Fmoc-amino acids to SWNTs. The maximum peak was observed at 300.3 ± 0.08 nm for G, 300.1 ± 0.3 nm for W and 300.3 ± 0.06 for C. All Fmoc derivatives had adsorption profiles fluctuating in various extents during 144 h (Figure 4B). This showed the dynamic behavior of the Fmoc derivatives that reversibly adsorbed and desorbed on the SWNT surface. C marked the maximum level for loading at the end of 24 h. The amounts of W and G interacting with the SWNT were relatively lower during the loading experiments. Even though the adsorption profiles of Fmoc derivatives did not reach a plateau at 144 h for all cases, the highest amounts of adsorption were consistently observed for C. This result seemed to conflict with the TGA data; but in fact, it confirmed the excessive stacking of C molecules suggested by the MD simulations. The washing process was not employed in the loading experiments, which revealed the multilayer coating formed by stacked C molecules on the nanotube surface.

**FIGURE 5:**
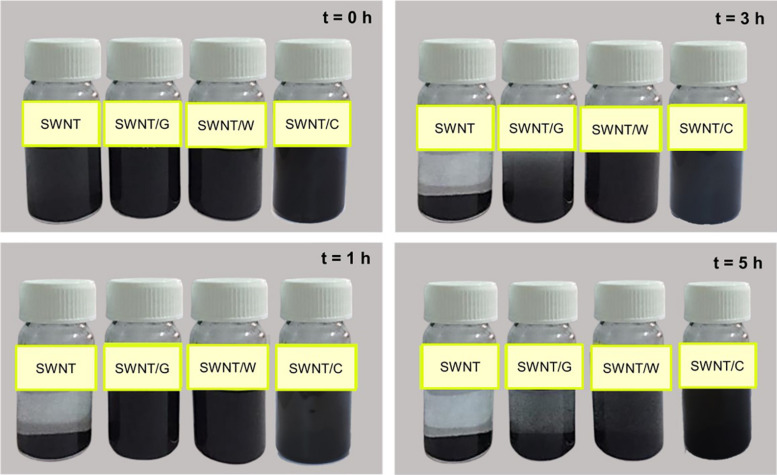
Distribution of SWNTs, SWNT/G, SWNT/W, and SWNT/C in water.

Once the Fmoc-amino acids are adsorbed on the SWNT surface, their polar groups in the amide backbone (-OH and -COOH) adopt surfactant-like properties. Additional interactions between the adsorbed and not adsorbed Fmoc-amino acids are expected to occur through π-π stacking and hydrogen bond interactions, as noted from MD simulations. To gain a more understanding of the coating of SWNTs, the water dispersion behaviors of the modified SWNTs with Fmoc-amino acids are illustrated in Figure 5. SWNT/C complexes displayed enhanced dispersion and more stability at least for 5 h, followed by SWNT/W. SWNT/G showed poor stability in the water medium. These results supported the interaction energy calculations in MD simulations highlighting C with the highest affinity to SWNT among the other Fmoc derivatives. The dynamic behavior of C molecules is also a strong indicator for water stability of C-coated SWNTs. Consequently, the relatively lower amount of C adsorbed on the SWNT, due to the reasons discussed above, was more effective for SWNT suspension in water. Therefore, based on this preliminary study, we expect that W and C will highly improve the suspension of hydrophobic SWNTs in the water when attached to PEG.

### Synthesis and Characterization of PEG-Coated SWNTs

**FIGURE 6:**
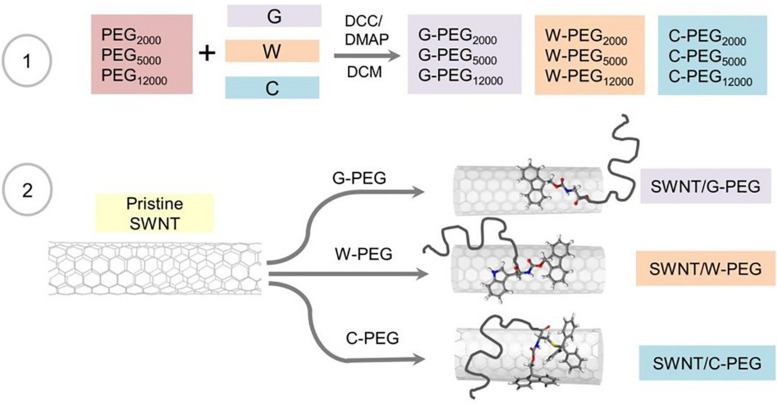
Schematic illustration of the reactions involved in the preparation of the PEGylated SWNTs.

Figure 6 summarizes the steps followed for the synthesis of Fmoc-amino acid-PEG coated SWNTs. First, PEGs with different molecular weights (Mw = 2,000, 5,000, 12,000 g.mol^–1^) were treated with Fmoc-amino acid (G, W, or C) in CH_2_Cl_2_ using the Steglich Esterification mechanism (Supplementary Figure 6). Then, the non-covalent functionalization of pristine SWNTs was achieved using G, W, and C functional PEGs via π-π interactions. We used the same amount of Fmoc-PEG chains (6.02 mol Fmoc-PEG per gram SWNT) to explore the effect of molecular weight of the linear PEG chain as well as the Fmoc-amino acid anchor.

**FIGURE 7:**
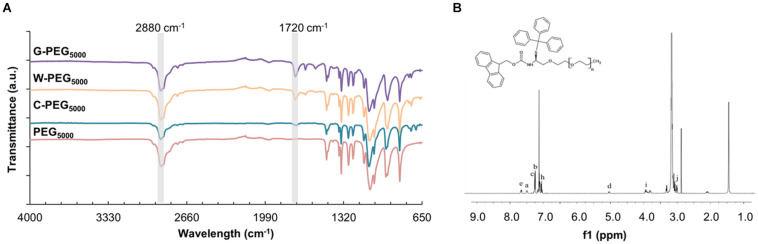
**(A)** FT-IR spectrum of G-PEG_5000_, W-PEG_5000,_ C-PEG_5000_, and PEG_5000_ **(B)** ^1^H NMR spectrum of C-PEG_5000._

Figure 7A shows the FT-IR spectra of PEG_5000_ – based samples as an example, while the other spectra are given in Supplementary Figure 7. We noted no remarkable difference between the FT-IR assignments of the Fmoc-PEG complexes. The characteristic peaks belonging to PEG repeating unit (O-CH_2_-CH_2_) appeared around 2,880 cm^–1^ in all three spectra (Zeng et al., 2014). Additionally, carbonyl stretching peaks of Fmoc units were spotted at 1,720 cm^–1^ (Tao et al., 2016). FT-IR analysis thus confirmed the formation of Fmoc-PEG complexes. To further support the FT-IR results, the formation of G-, W-, and C-PEGs were analyzed using H-NMR spectroscopy. Figure 7B illustrates the NMR spectrum of C-PEG_5000_ as an example, where the other NMR spectra are given in the Supplementary Figures 8–10. Signals at 3.6 ppm indicated the methylene protons of the PEG backbone, whereas the aromatic Fmoc and C groups appeared in the range of 7–8 ppm.

**FIGURE 8:**
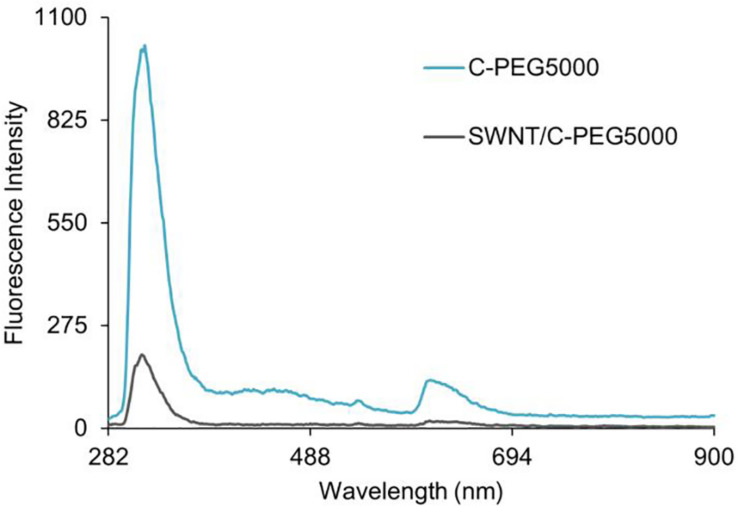
Fluorescence spectra of C-PEG_5000_ and SWNT/C-PEG_5000_.

The quenching of the fluorescence spectra was used to characterize the existence of the π-π interactions between the Fmoc-amino acid-PEG chains and SWNTs, as was previously shown for various carbon nanotube-based complexes (Sen et al., 2012; Zeng et al., 2014; Tao et al., 2016). Here, the intermolecular π-π stacking between aromatic rings can effectively cause fluorescence quenching through energy transfer (Sen et al., 2012), also in the presence of long hydrophilic PEG chains (Suk et al., 2016). Figure 8 represents the fluorescence spectra of C-PEG_5000_ and SWNT/C-PEG_5000_ with maximum fluorescence intensity at 265 nm, while the spectra for the other samples are given in Supplementary Figures 11–13. Each fluorescence spectrum was determined according to maximum absorbance measured by the UV-vis spectrophotometer. For the SWNT/Fmoc-PEG complex, the fluorescence intensities of 319, 318, and 265 nm were noted for W, G, and C groups, respectively.

**FIGURE 9:**
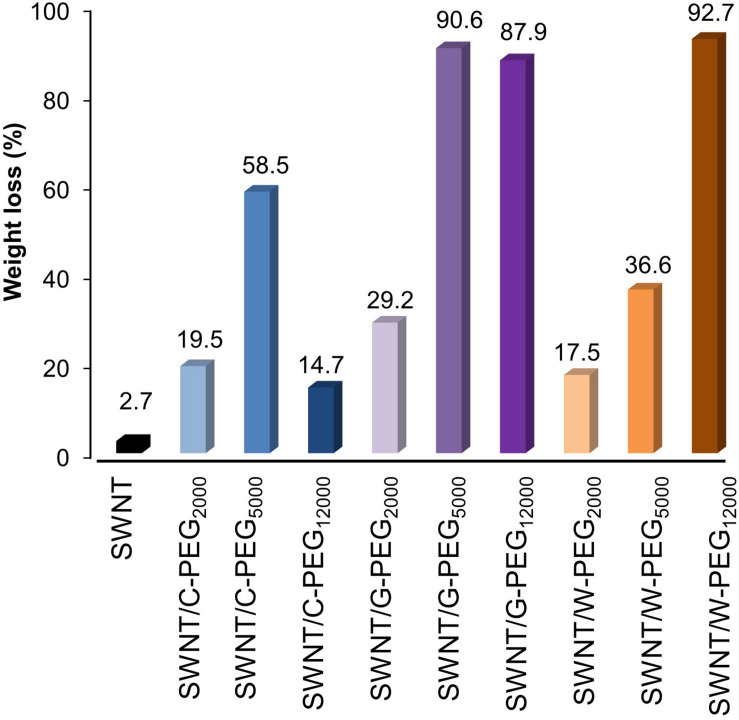
Thermogravimetric results of bare SWNTS and SWNTs coated with G-PEGs, W-PEGs, and C-PEGs.

Thermal gravimetric analysis was used to quantify the amount of surface coating of SWNTs by Fmoc-PEG chains (Supplementary Figure 14). The largest mass decrease at about 410°C was caused by the thermal decomposition of Fmoc-amino acid-PEG complexes due to their lower thermal stability compared to SWNTs. The weight loss for all samples was calculated at 800°C to make sure organic matter was completely removed. Figure 9 shows that all PEG samples can surface coat the SWNTs, using π-π interactions between Fmoc derivatives and SWNT, as well as van der Waals interactions between PEG chains and SWNT, as was previously reported (Akkuş and Kürkçüoğlu Levitas, 2019). As a general trend, with an increase in the molecular weight of PEG, the weight loss increases. However, the weight loss of C-PEG_12000_ was intriguingly smaller comparing to C-PEG_2__000_ and C-PEG_5__000_. Here, at one end of long PEG_12000_ chains, C molecules have multiple aromatic groups that are linked by flexible linkers. These can make an extensive amount of π-π stackings (see Figure 3), which in turn may prevent their adsorption on the nanotube surface and/or cavity.

**FIGURE 10:**
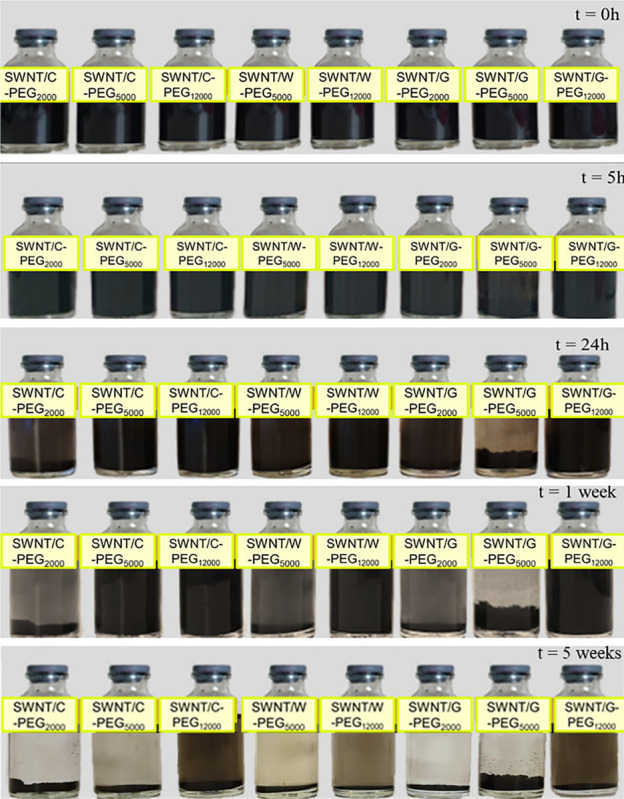
Distribution of SWNT/G-PEG_2000_, SWNT/G-PEG_5000_, SWNT/G-PEG_12000_, SWNT/W-PEG_5000_, SWNT/W-PEG_12000_, SWNT/C-PEG_2000_, SWNT/C-PEG_5000_, and SWNT/C-PEG_12000_ in water.

Finally, the water dispersion behavior of Fmoc-PEG functionalized SWNTs were monitored for 5 weeks (Figure 10). All samples were well dispersed in deionized water. No precipitation was observed in any sample at the end of 5 h. These complexes outperformed the SWNT/Fmoc-derivatives (Figure 5) and underlined the success of incorporating PEG chains into the non-covalent coating. After 24 h, complexes with PEG_2000_ and PEG_5000_ precipitated in water, except SWNT/C-PEG_5000_. A 1 week later, SWNT/C-PEG_5000_, SWNT/C-PEG_12000_, SWNT/G-PEG_12000_, and SWNT/W-PEG_12000_ were still stable in water. At the end of 5 weeks, although SWNT/C-PEG_12000_, SWNT/G-PEG_12000_, and SWNT/W-PEG_12000_ did not completely settle in water, SWNT/C-PEG_12000_ was the most suspended among them. We concluded that longer PEG chains (PEG_12000_) provide effective coatings for SWNT non-covalent functionalization regardless of their Fmoc-amino acid ends. Here, PEG12000 functionalized SWNTs did not precipitate in water, since long chains wrap around themselves while interacting with the SWNT surface through van der Waals forces. Besides, Fmoc derivatives of the long PEG chains were adsorbed on the nanotube surface with interaction energy around −35 kcal.mol^–1^ (C) to −25 kcal.mol^–1^ (G). Among the Fmoc derivatives, Fmoc-C provided the highest stability and good water dispersion properties to pristine SWNT walls, even small amounts were used for coating. The success of C-PEG_500__0_ can also be explained by the high interaction energy between C and the nanotube wall. Accordingly, W-PEG_500__0_ and G-PEG_500__0_ were expected to detach from the SWNT surface in water, as a result, sedimentation of the nanocarriers was observed after a certain period.

### *In vitro* Cell Viability Assay

**FIGURE 11:**
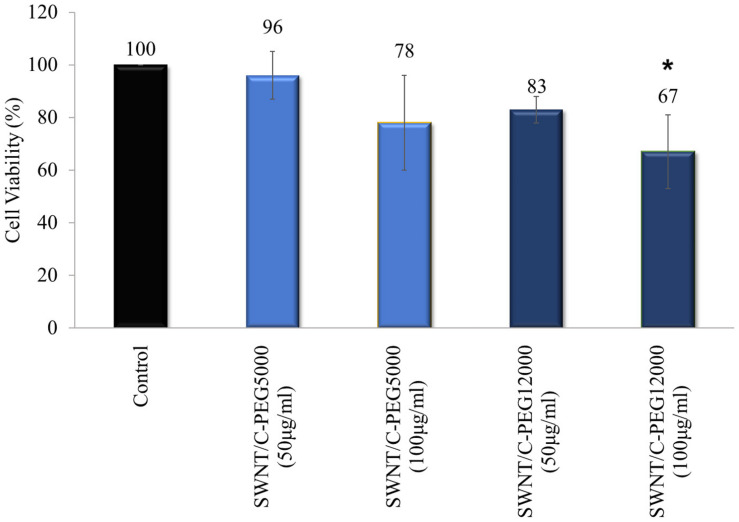
Cell viability results for SWNT/C-PEG_5000_, and SWNT/C-PEG_12000_. Cells were treated with two concentrations of SWNT-based samples. The percentages of cell viability were normalized to control cells. These results are the mean of triplicate experiments. The results were given as mean ± SD. *p < 0.05 compared to the control cells.

MTT assay was performed to detect the effect of non-covalently coated SWNTs on the viability of human dermal fibroblast cells. Fibroblast cells were treated with SWNT/C-PEG_5000_ and SWNT/C-PEG_12000_ in two concentrations for 24 h. SWNT/C-PEG_12000_ was subjected to MTT assays since it had the longest water suspension time. The SWNT/C-PEG_5000_ sample had also a better dispersion behavior among the other shorter PEG chain samples, up to 1 week. Figure 11 indicates that 50 μg/ml of both SWNT/C-PEG_5000_ and SWNT/C-PEG_12000_ had low cytotoxic effect on the cells maintaining 96 and 83% viability, respectively. Even at a higher concentration of 100 μg/ml, the cell viability was noted as 78% for the SWNT/C-PEG_5000_ samples. SWNT/C-PEG12000 had a higher cytotoxicity (67% cell viability) at 100 μg/ml concentration.

As indicated by the two-way ANOVA, a statistically significant (*p* < 0.05) difference was found between this group and control group in view of cell viability. Also, the process included the evaluation of the material type and applied dose influence on cell viability. The most noteworthy effect on the cell viability was shaped by the applied dose of material, while material type had less impact.

SWNT/C-PEG_5000_ did not show any toxic effect on the fibroblast cells, therefore had the best cell viability performance. This result also supported its good dispersion in water.

## Conclusion

Here, we employed a convenient and efficient synthesis protocol for SWNT-based nanoparticles coated with hydrophilic PEG chains. Non-covalent coating of pristine SWNTs was performed to improve their dispersion in water, hence their biocompatibility while preserving the intrinsic properties of the nanotubes. With this purpose, long hydrophilic Fmoc-PEG chains were conjugated to SWNTs through π-π interactions. The MD simulations highlighted two Fmoc-amino acids, cysteine, and tryptophan with higher interaction energies with SWNT (6,6). Computational results along with TGA and water suspension studies motivated us to synthesize PEG chains with cysteine, tryptophan, and glycine functionalities, where the latter was the non-aromatic control. We demonstrated the successful synthesis of Fmoc-amino acid-PEG complexes, which were further employed for the surface coating of SWNTs. Water suspension experiments, mimicking the biological settings, revealed that Fmoc-cysteine attached PEG chains stabilized hydrophobic SWNTs for up to 5 weeks. We then showed the low cytotoxicity of SWNTs coated with Fmoc-cysteine attached PEG chains. Incorporation of PEG chains to Fmoc-amino acids and their usage to non-covalently coat SWNTs for dispersion in an aqueous environment are reported for the first time to our knowledge. Overall, Fmoc-PEG-coated SWNT nanoparticles developed in this study promise as effective nanocarriers for various therapeutic agents, while enabling theranostic applications including imaging and active targeting for different types of diseases.

## Data Availability Statement

The raw data supporting the conclusions of this article will be made available by the authors, without undue reservation.

## Author Contributions

FG designed the work. YY and ÖG-Y synthesized and characterized the carbon nanotube composites. MM supervised the reaction conditions for the functionalization of SWNTs. SK carried the molecular dynamics simulations and OK evaluated the results. SBo synthesized the carbon nanotubes. NK evaluated the data for the synthesis of SWNTs. EB synthesized the Fmoc-PEG complexes. GH analyzed the findings. SBa performed the MTT assays. YY, ÖG-Y, SK, SBo, MM, and EB wrote the original draft. FG and OK edited the final draft. All authors contributed to the article and approved the submitted version.

## Conflict of Interest

The authors declare that the research was conducted in the absence of any commercial or financial relationships that could be construed as a potential conflict of interest. The reviewer MC declared a shared affiliation, with no collaboration, with one of the authors FG to the handling editor at the time of the review.
