# Supplementary material for: Fmoc-PEG Coated Single-Wall Carbon Nanotube Carriers by Non-covalent Functionalization: An Experimental and Molecular Dynamics Study
Source: Front Bioeng Biotechnol. 2021 May 14;9:648366. doi: 10.3389/fbioe.2021.648366 (PMC8160473; doi:10.3389/fbioe.2021.648366)
Supplement: Supplementary file 1 [file Data_Sheet_1.PDF]

Supplementary Material

Fmoc-PEG Coated Single-Wall Carbon Nanotube Carriers by Non-Covalent Functionalization: An Experimental and Molecular Dynamics Study

Yesim Yeniyurt[1, †], Sila Kilic[1, †], Ö. Zeynep Güner-Yılmaz[1, †], Serdar Bozoglu[2], Mehdi Meran[1,3], Elif Baysak[4], Ozge Kurkcuoglu[1], Gurkan Hizal[4], Nilgun Karatepe[2], Saime Batirel[5], F. Seniha Güner[1,6]*

[1]Department of Chemical Engineering, Istanbul Technical University, Maslak 34469 Istanbul, Turkey

[2]Energy Institute, Renewable Energy Division, Istanbul Technical University, Maslak 34469 Istanbul, Turkey

[3]Bioengineering Department, Faculty of Engineering and Natural Sciences, Üsküdar University, Üsküdar 34662, Istanbul, Turkey

[4]Department of Chemistry, Istanbul Technical University, Maslak 34469 Istanbul, Turkey

[5]Department of Medical Biochemistry, School of Medicine, Marmara University, Maltepe 34854, Istanbul, Turkey

[6]Sabanci University Nanotechnology Research and Application Center (SUNUM), Sabanci University, 34956 Istanbul, Turkey

[†]These authors have contributed equally to this work and share first authorship

*** Correspondence:**
Corresponding Author
guners@itu.edu.tr

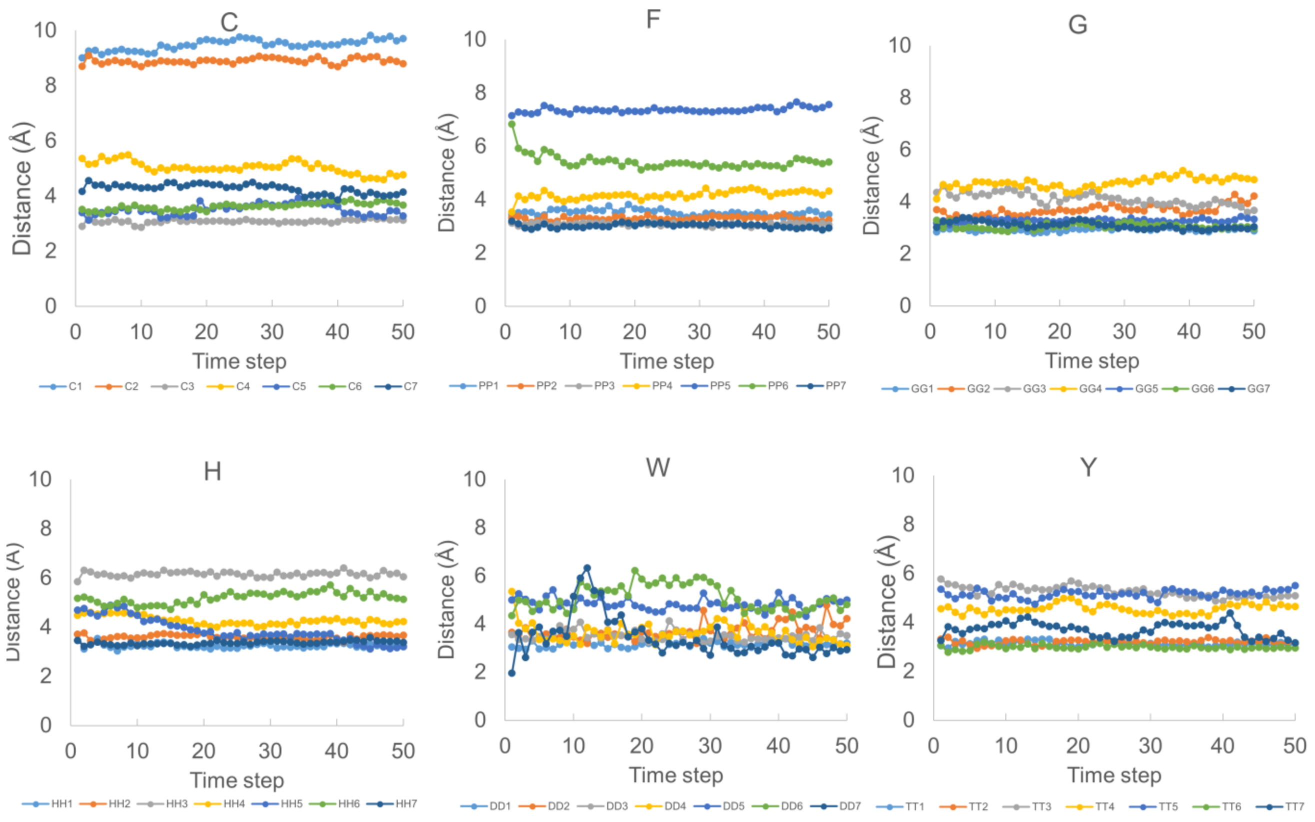

Figure S1. Evolution of distance between Fmoc and SWNT surface for 1 ns long MD quench simulations

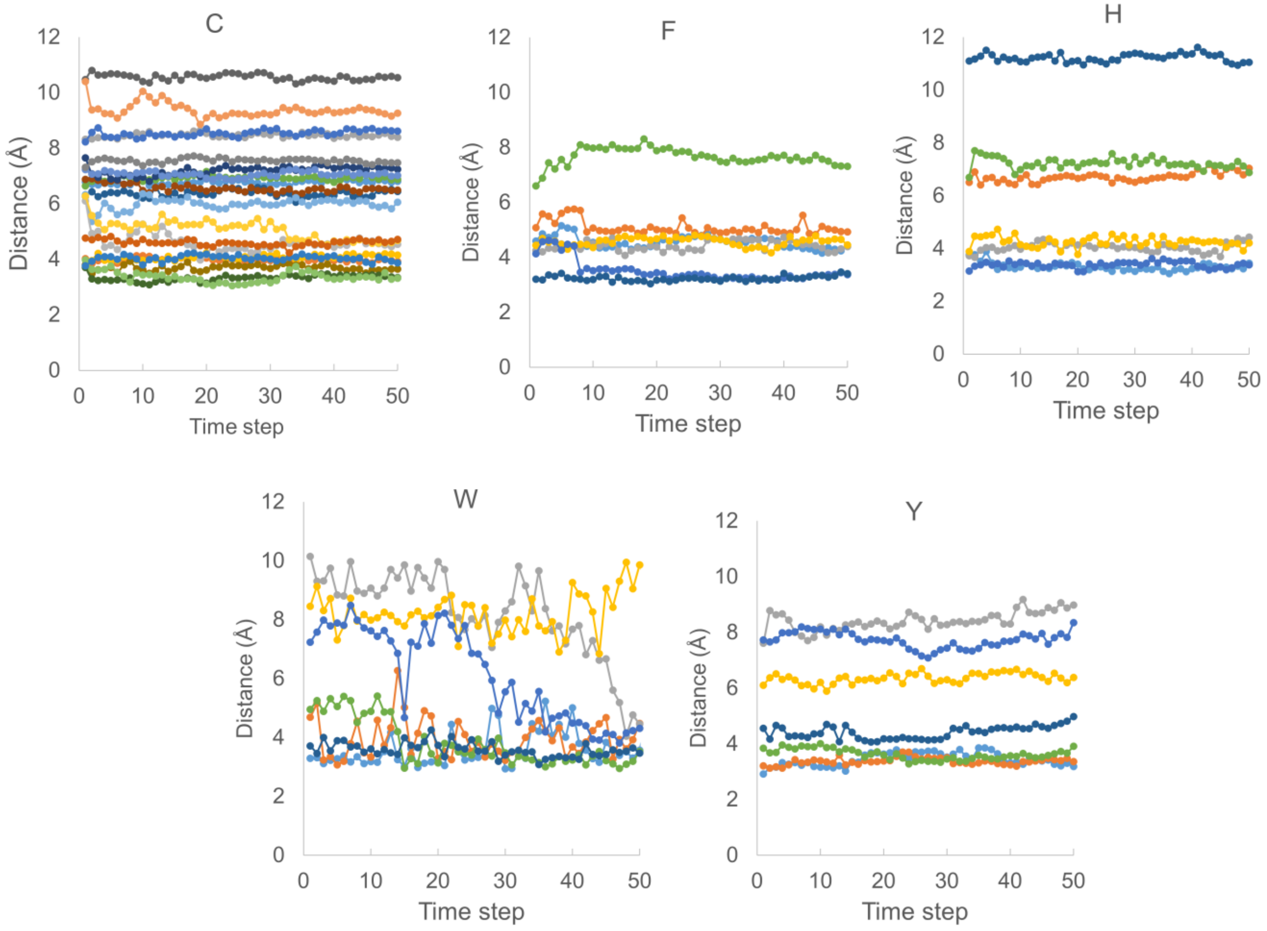

Figure S2. Evolution of distance between aromatic side chain and SWNT surface for 1 ns long MD quench simulations

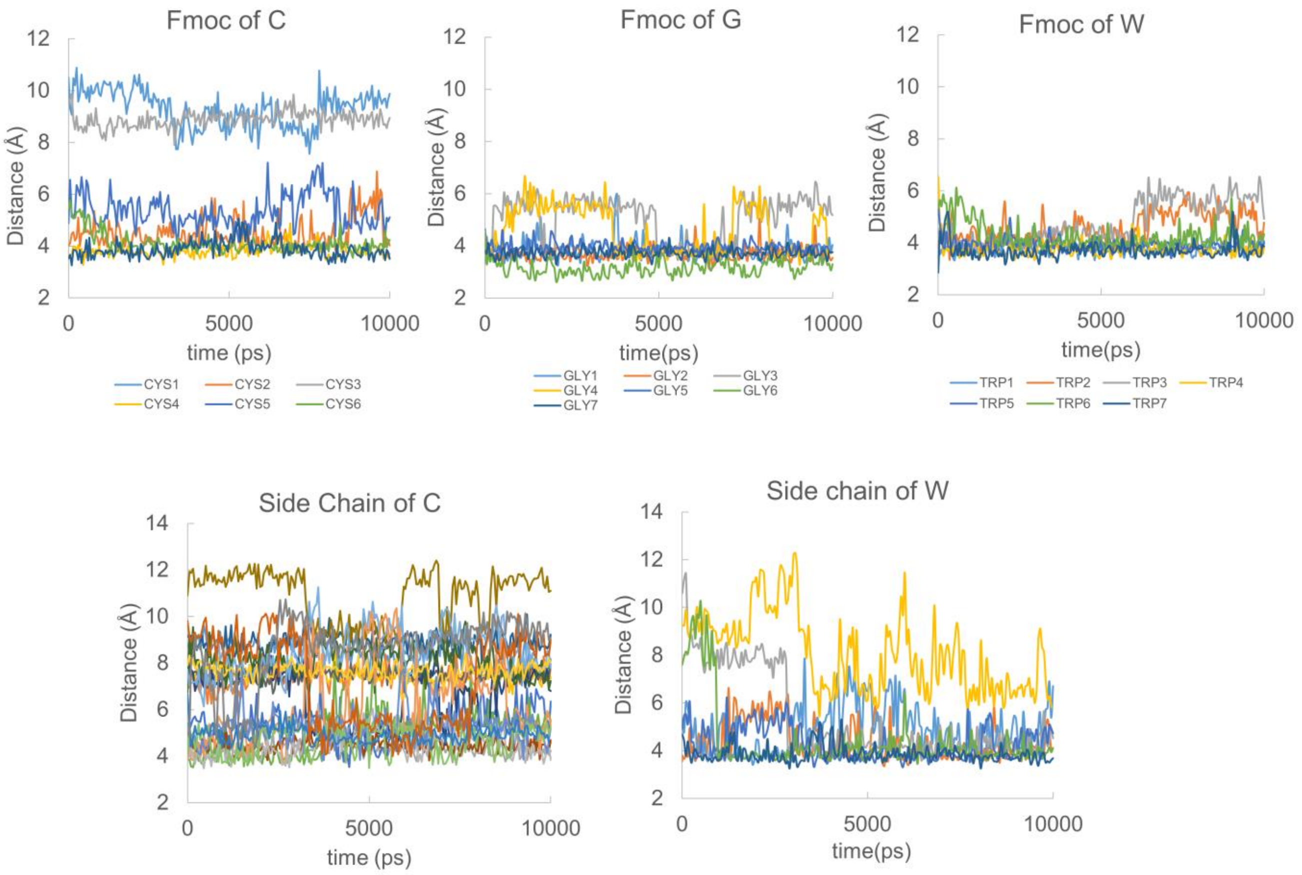

Figure S3. Evolution of distance between Fmoc and SWNT surface and aromatic side chain, and SWNT for 10 ns long MD simulations

A.

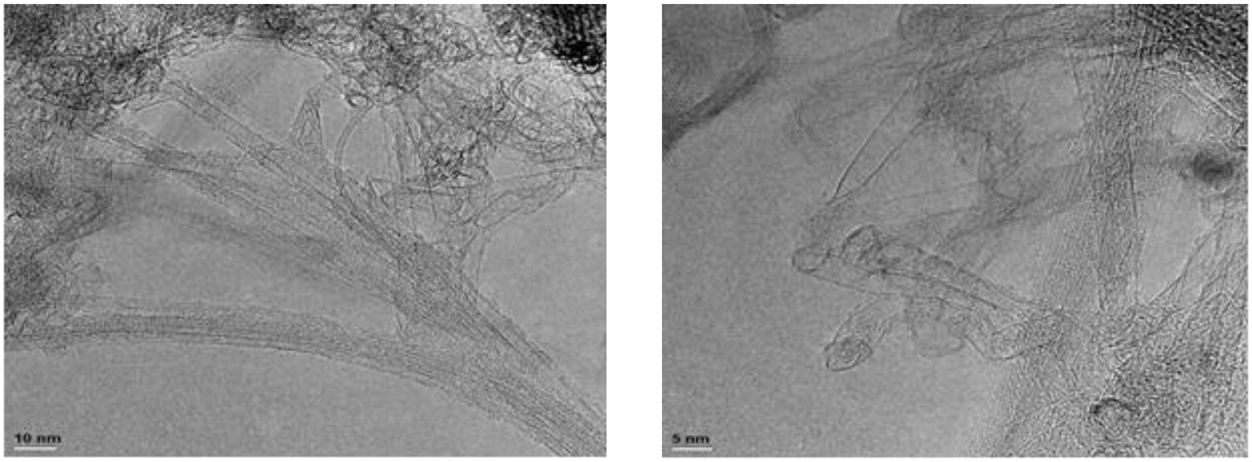

B.

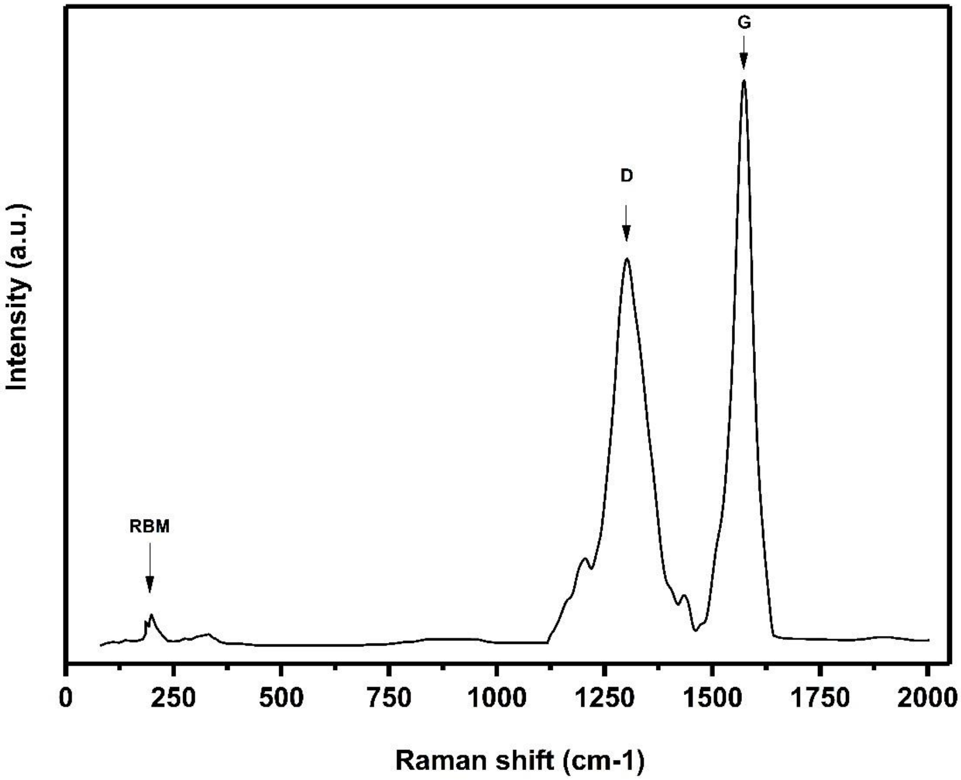

Figure S4. A. TEM images and, **B.** Raman spectra of SWNT

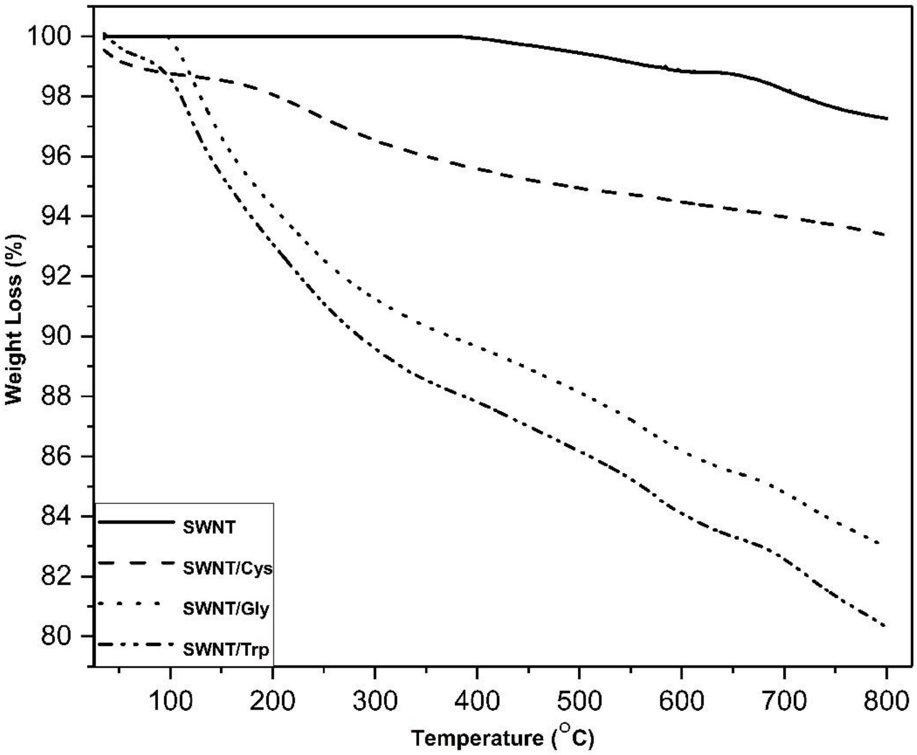

Figure S5. TGA results for Fmoc-amino acid coated SWNTs

A.

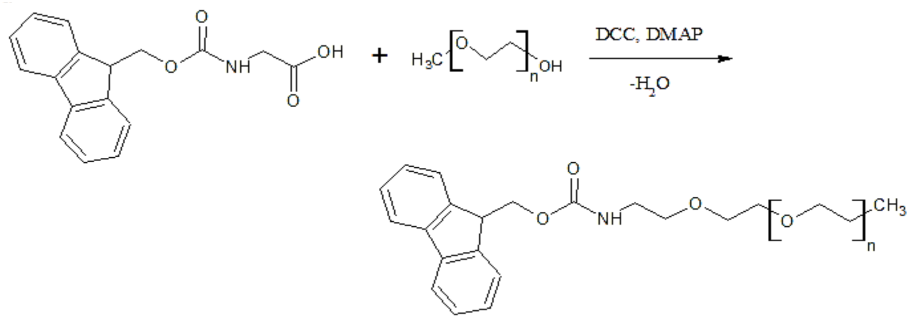

B.

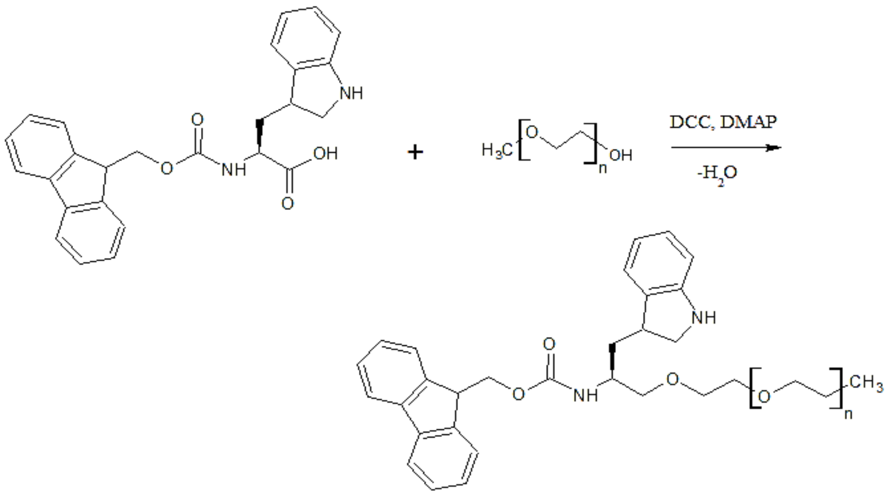

C.

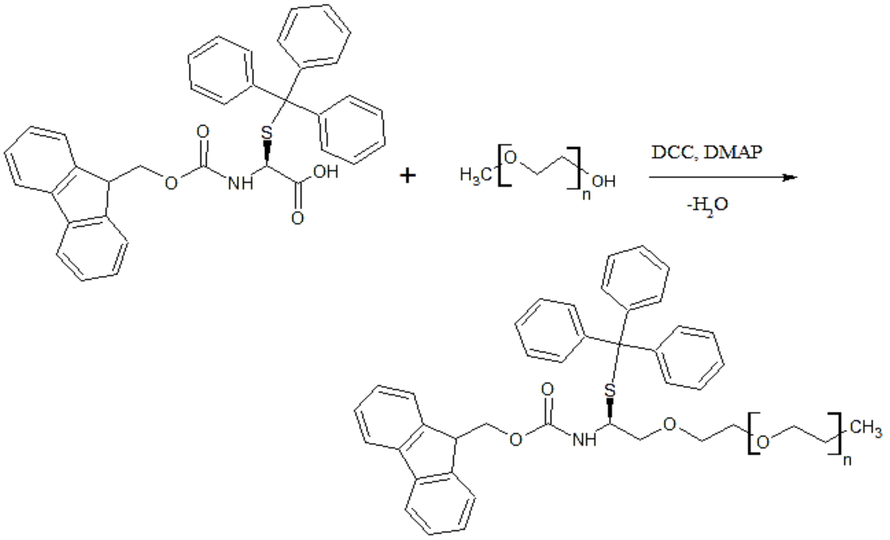

Figure S6. Synthesis of **A.** G, **B.** W, and **C.** C functionalized PEGs.

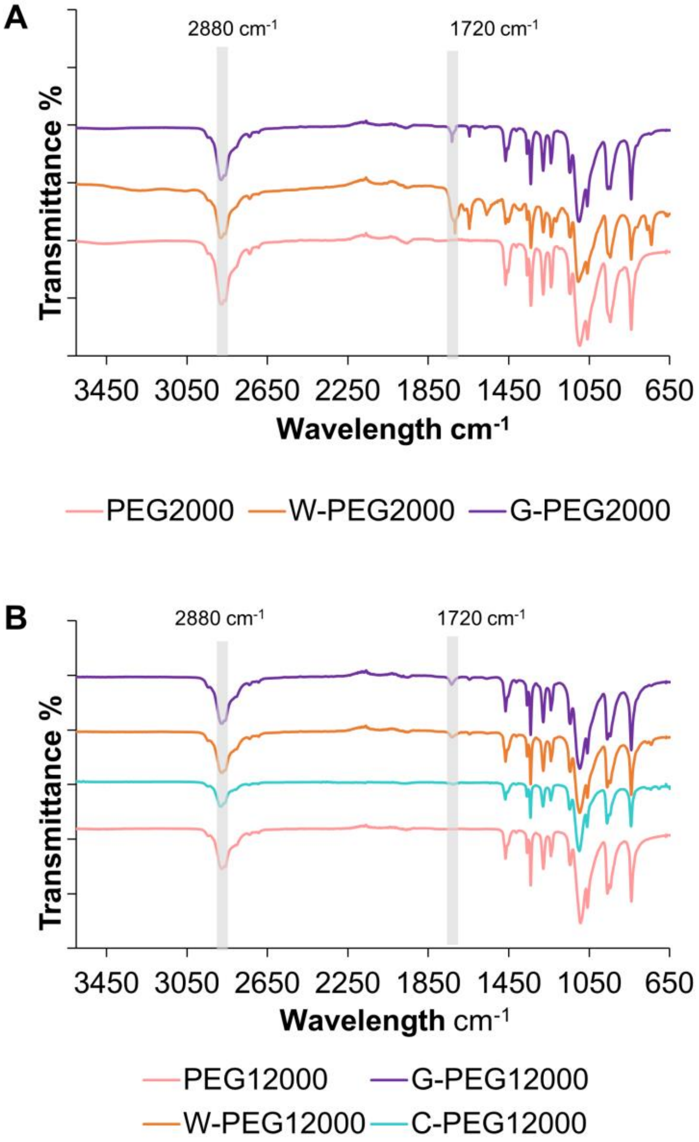

Figure S7. A. FT-IR spectrum of G-PEG$_{2000}$, W-PEG$_{2000}$, C-PEG$_{2000}$ and PEG$_{2000}$. **B.** FT-IR spectrum of G-PEG$_{12000}$, W-PEG$_{12000}$, C-PEG$_{12000}$ and PEG$_{12000}$

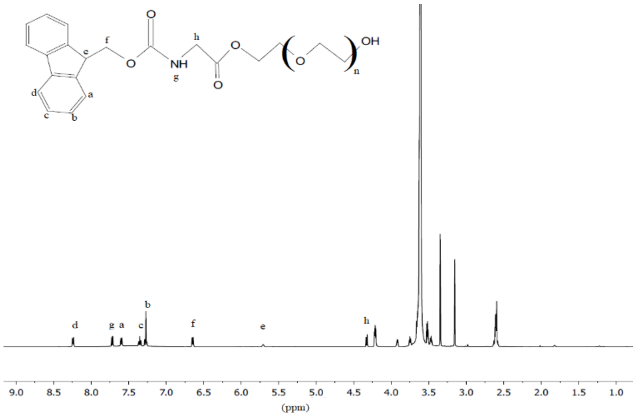

A.

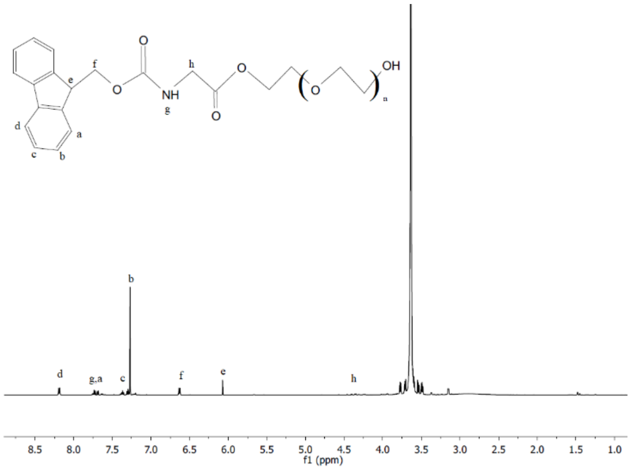

B.

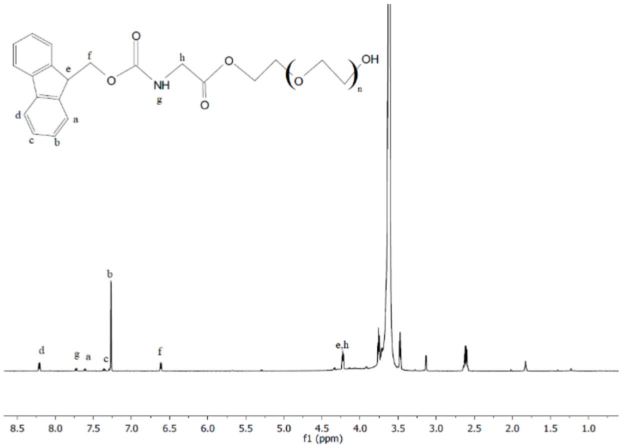

C.

Figure S8. ^{1}H NMR spectrum of **A.** G-PEG$_{2000}$, **B.** G-PEG$_{5000}$ and **C.** G-PEG$_{12000}$.

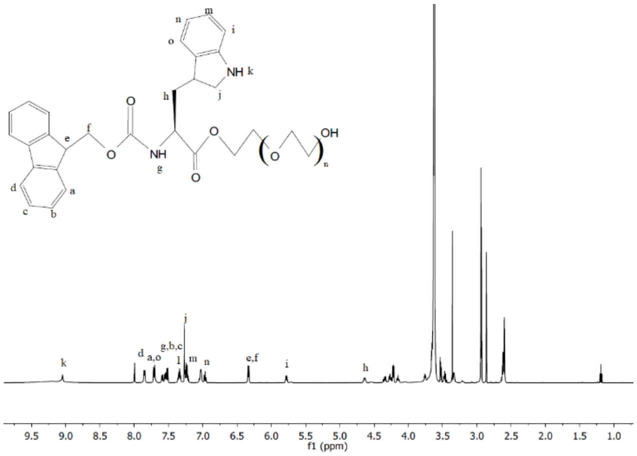

A.

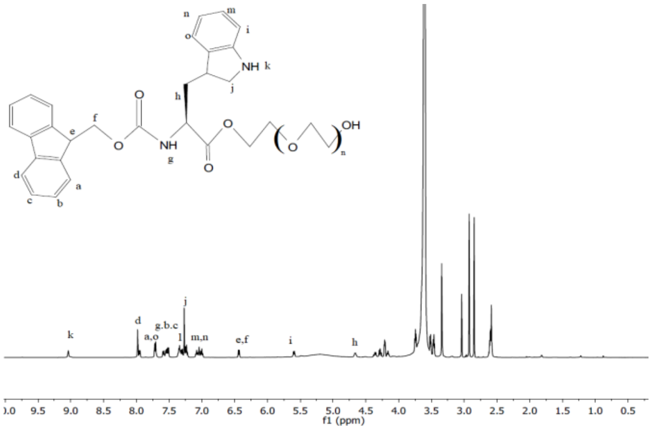

B.

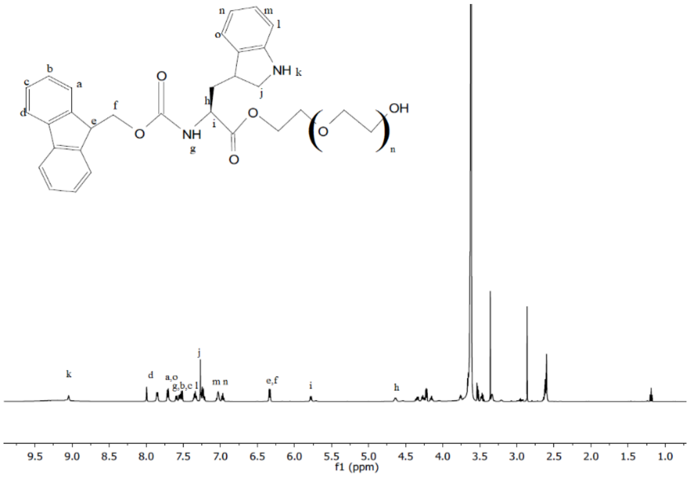

C.

Figure S9. ^{1}H NMR spectrum of **A.** W-PEG$_{2000}$, **B.** W-PEG$_{5000}$, and **C.** W-PEG$_{12000}$.

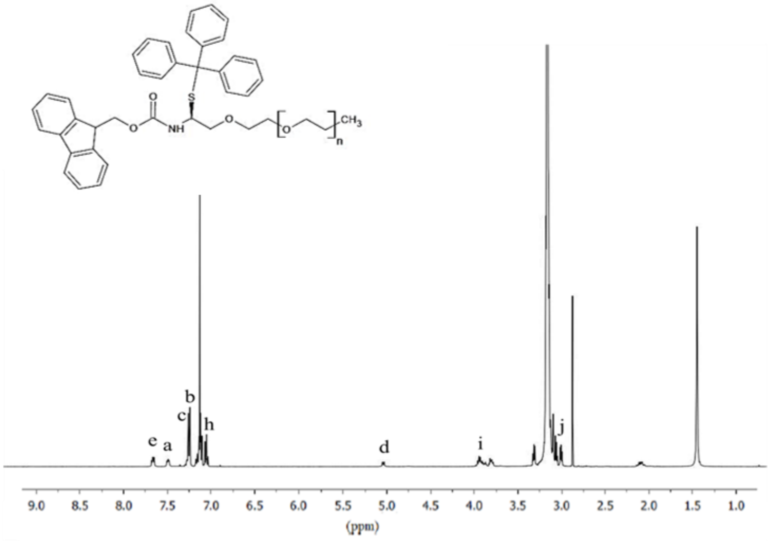

A.

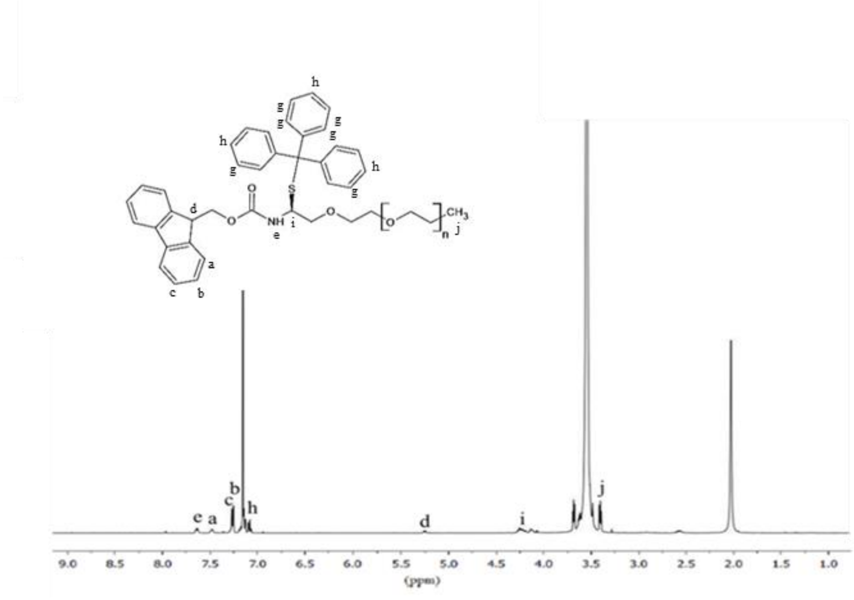

B.

Figure S10. ^{1}H NMR spectrum of **A.** C-PEG$_{2000}$ and **B.** C-PEG$_{12000}$.

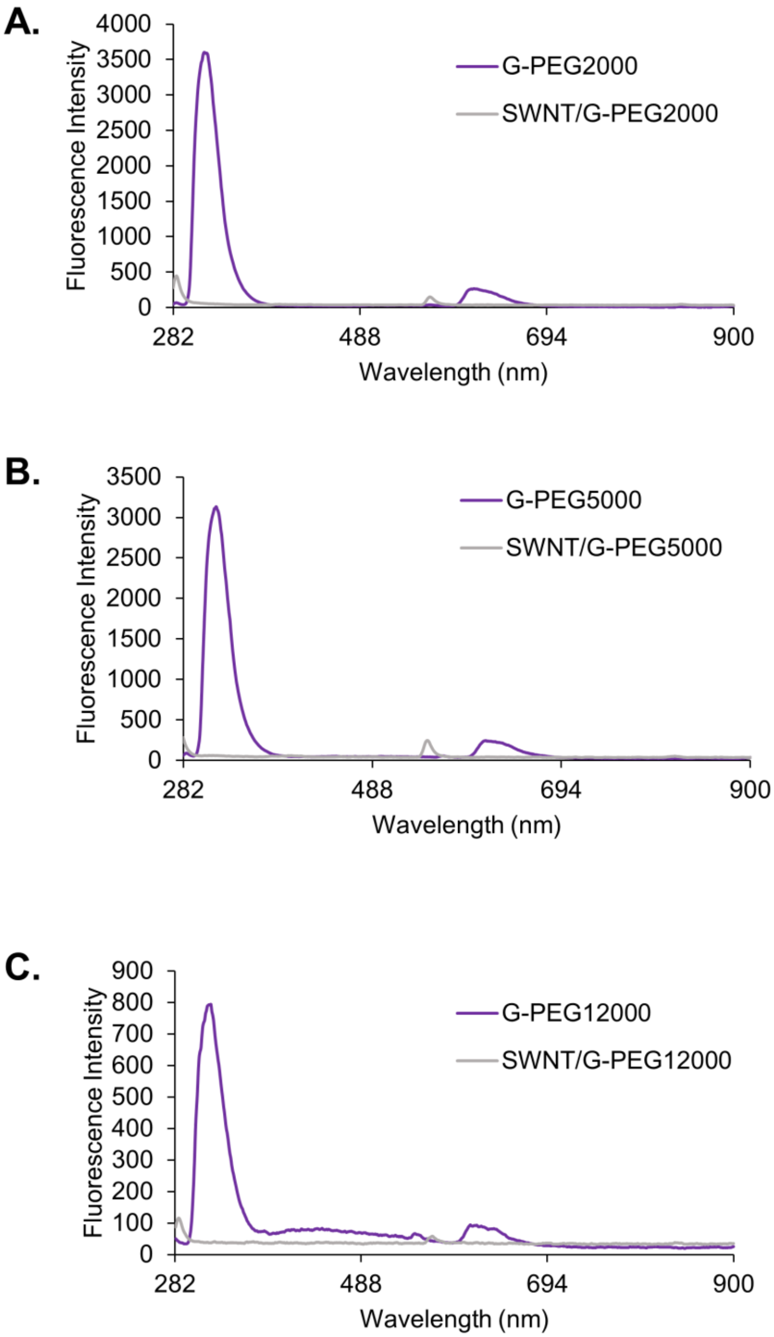

Figure S11. Fluorescence spectra of **A.** G-PEG2000 and SWNT/G-PEG_{2000}, **B.** G-PEG_{5000} and SWNT/G-PEG_{5000}, and **C.** G-PEG_{12000} and SWNT/G-PEG_{12000}.

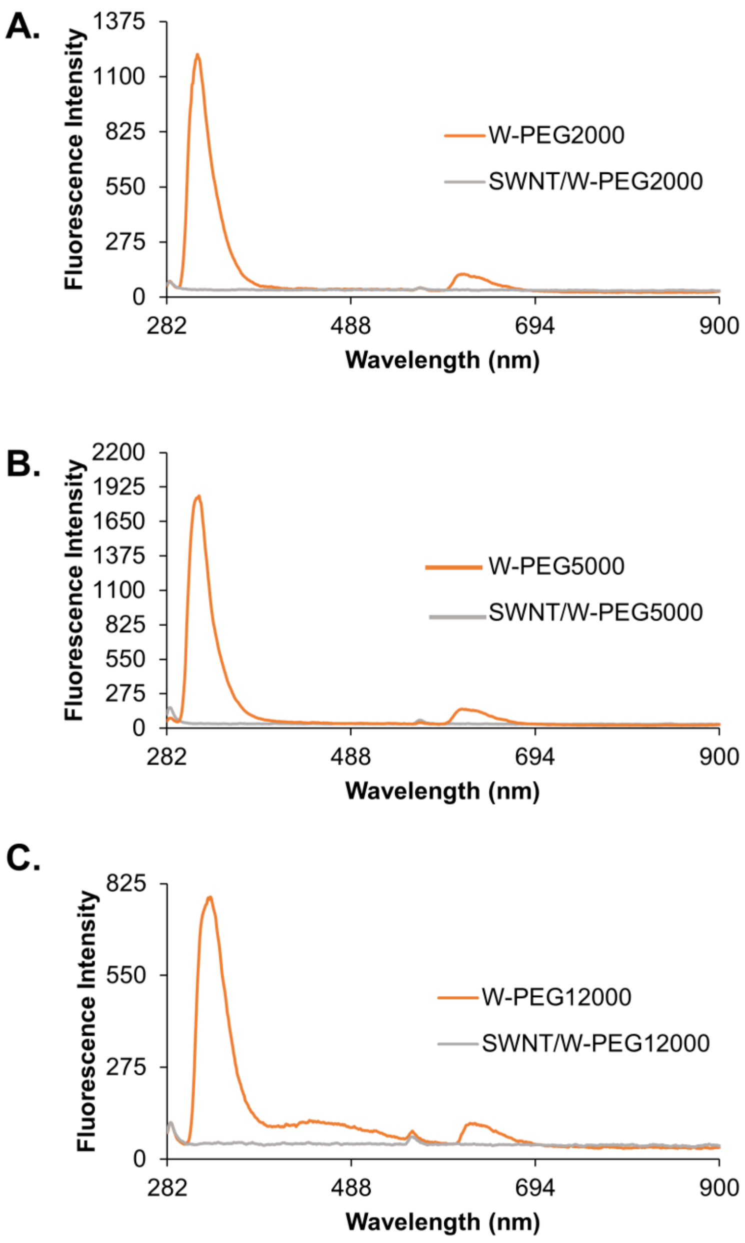

Figure S12. Fluorescence spectra of **A.** W-PEG$_{2000}$ and SWNT/W-PEG$_{2000}$, **B.** W-PEG$_{5000}$ and SWNT/W-PEG$_{5000}$, **C.** W-PEG$_{12000}$ and SWNT/W-PEG$_{12000}$.

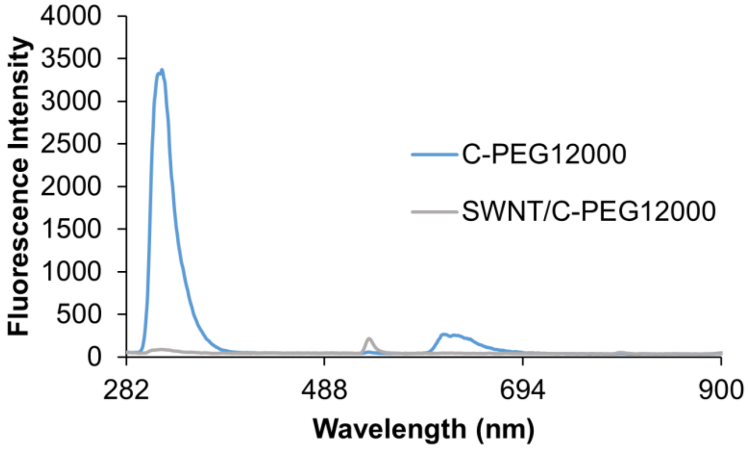

Figure S13. Fluorescence spectra of C-PEG_{12000} and SWNT/C-PEG_{12000}.

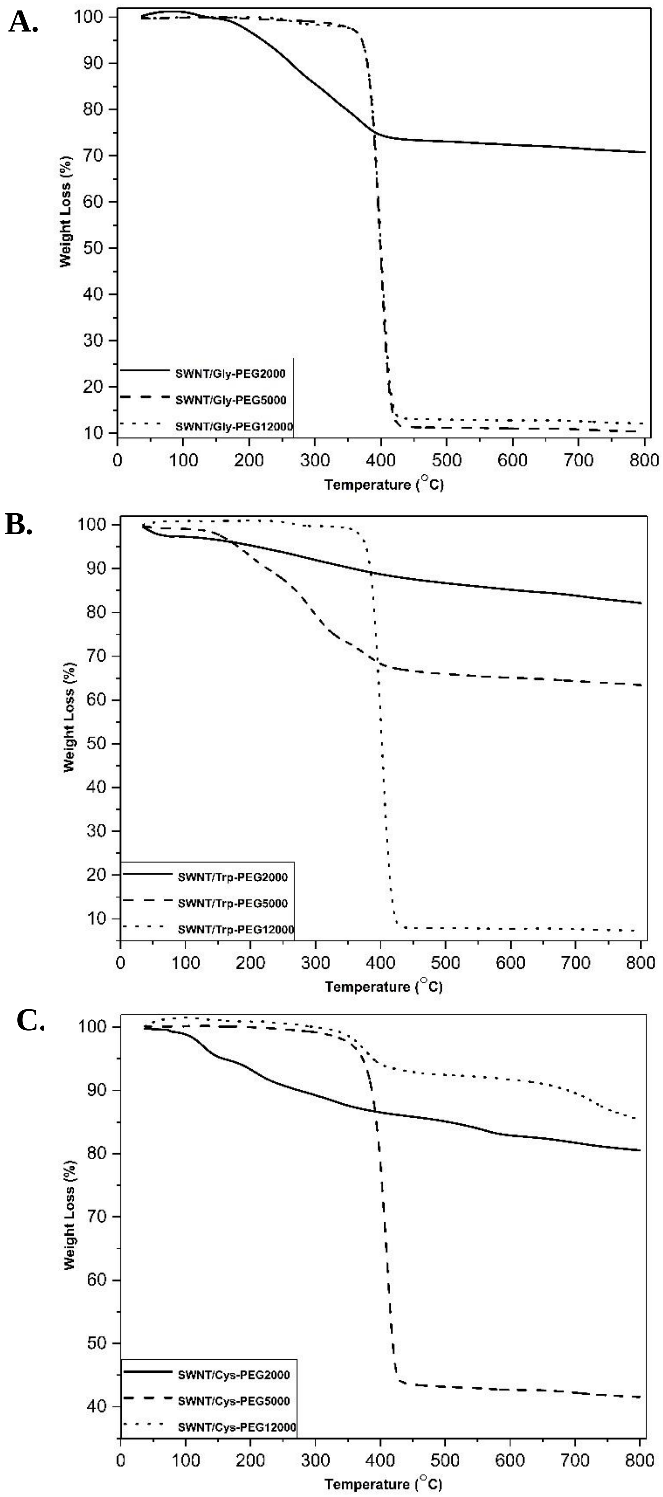

Figure S14. TGA results for **A.** G-PEG coated SWNTS, **B.** W-PEG coated SWNTs and **C.** C-PEG coated SWNTS
